# Low-dose radiation induces unstable gene expression in developing human iPSC-derived retinal ganglion organoids

**DOI:** 10.1038/s41598-023-40051-6

**Published:** 2023-08-09

**Authors:** Mari Katsura, Yoshihiro Urade, Hiroko Nansai, Mika Kobayashi, Akashi Taguchi, Yukiko Ishikawa, Tomohiro Ito, Hisako Fukunaga, Hideto Tozawa, Yoko Chikaoka, Ryo Nakaki, Akinobu Echigo, Takahide Kohro, Hideko Sone, Youichiro Wada

**Affiliations:** 1 Isotope Science Center, The University of Tokyo, Tokyo, Japan; 2 Reiwa Eye Clinic, Hatsukaichi, Hiroshima Japan; 3 Graduate School of Medicine and Faculty of Medicine, The University of Tokyo, Tokyo, Japan; 4 Center for Health and Environmental Risk Research, National Institute for Environmental Studies, Tsukuba, Ibaraki Japan; 5 Graduate School of Science, The University of Tokyo, Tokyo, Japan; 6 Rhelixa Co., Ltd, Tokyo, Japan; 7 Tomy Digital Biology Co., Ltd, Tokyo, Japan; 8 Department of Clinical Informatics, Jichi Medical University, Shimotsuke, Tochigi Japan; 9 Environmental Health and Prevention Research Unit, Yokohama University of Pharmacy, Yokohama, Japan; 10 Research Center for Advanced Science and Technology, The University of Tokyo, Tokyo, Japan

**Keywords:** Cell biology, Computational biology and bioinformatics, Developmental biology, Molecular biology, Neuroscience, Stem cells, Environmental sciences, Environmental social sciences, Health occupations, Neurology, Risk factors, Energy science and technology

## Abstract

The effects of low-dose radiation on undifferentiated cells carry important implications. However, the effects on developing retinal cells remain unclear. Here, we analyzed the gene expression characteristics of neuronal organoids containing immature human retinal cells under low-dose radiation and predicted their changes. Developing retinal cells generated from human induced pluripotent stem cells (iPSCs) were irradiated with either 30 or 180 mGy on days 4–5 of development for 24 h. Genome-wide gene expression was observed until day 35. A knowledge-based pathway analysis algorithm revealed fluctuations in Rho signaling and many other pathways. After a month, the levels of an essential transcription factor of eye development, the proportion of paired box 6 (PAX6)-positive cells, and the proportion of retinal ganglion cell (RGC)-specific transcription factor POU class 4 homeobox 2 (POU4F2)-positive cells increased with 30 mGy of irradiation. In contrast, they decreased after 180 mGy of irradiation. Activation of the “development of neurons” pathway after 180 mGy indicated the dedifferentiation and development of other neural cells. Fluctuating effects after low-dose radiation exposure suggest that developing retinal cells employ hormesis and dedifferentiation mechanisms in response to stress.

## Introduction

The central nervous system of the foetus is very sensitive to ionizing radiation^1^. The International Commission on Radiological Protection (ICRP) predicts the threshold dose of ionizing radiation that causes abnormal neural development in the foetus to be 50–250 mGy^2^. Moreover, in the adult hippocampus, there are neural progenitor cells that are sensitive to irradiation. Cognitive impairments in brain cancer patients that occur after irradiation treatment are a serious problem^3^. As is the case for the increased medical use of ionizing radiation^4^, environmental radioactive pollution also presents additional challenges to human health^5^.

The risk of normal tension glaucoma (NTG) was elevated in atomic bomb survivors^6^. In patients with NTG, progressive loss of retinal ganglion cells (RGCs) decreases retinal thickness, resulting in subsequent visual field loss^7^, and it is one of the most common causes of blindness in elderly individuals. Müller glia cells are being studied as cells that may act as backup stem cells when retinal ganglion cells are damaged^8^. Although fish retinas have the ability to dedifferentiate and regenerate, such ability has not been found in mammals. Therefore, understanding the differentiation and dedifferentiation mechanisms of human retinal ganglion cells under stress not only deepens the understanding of the health effects from low-dose radiation exposure but also provides insights to elucidate the pathogenesis of eye diseases for which treatment methods have not been established.

Previously, we examined the effects of low-dose radiation on human gene regulation profiles in neural progenitor cells using microarray analysis. Consequently, we found that inflammation and cell junction pathways changed after exposure to less than 100 mGy irradiation^9^. The study revealed the effect just after exposure, showing that exposure to 31 mGy affected a few pathways related to the interferon pathway and insulin-like growth factor (IGF)^9^. Altered inflammation-associated gene expression may affect neuronal differentiation. Even less than 100 mGy of radiation can induce epigenetic changes^10^. Although many reports have documented the effects of low-dose radiation on neurogenesis in the brain^11–13^, there is currently no evidence suggesting that such low levels of radiation exposure have any impact on retinogenesis.

This is the first report revealing the effects of low-dose radiation on human retinal development. In this study, we used transcriptome analyses on developing retinal cells derived from human induced pluripotent stem cells (iPSCs). To address the varied responses of numerous genes to two different low-dose radiation levels, we employed an informatics-based strategy to identify significantly altered biological pathways. After a month, 30 and 180 mGy of irradiation induced different effects on retinal organoid development.

## Results

### RGCs from human iPSCs for genomic analysis

We developed neuronal organoids, including RGCs from human iPSCs, to assess the effects of low-dose irradiation. Phase-contrast microscopy (Supple Fig. S1a) indicated time-dependent morphological changes in the embryonal body formed from human iPSCs, which corresponded to previous reports^14, 15^. Retinal development was evaluated by the expressions of three marker genes specific for the eye and/or retina (Supple Fig. S1b); retinal homeobox gene (*RAX*), which is expressed in the retinal progenitor and stem cells, is essential for retinal development^16^; paired box 6 (*PAX6)*, which is essential for eye development, is a candidate gene of congenital aniridia^17^; and POU class 4 homeobox 2 (*POU4F2/BRN3B*), which is expressed in RGCs^18^, suppresses the differentiation of other retinal cell types^19^. The expression level of *RAX* started to increase on day 3, peaked on day 17, and gradually decreased on day 35. *PAX6* expression levels increased on day 3, peaked on day 25, and were maintained at a high level on day 35. *POU4F2* was expressed at a low level until day 17 and markedly increased from day 25 to 35. On day 35, POU4F2 was detected in the nuclei of most cells (Supple Fig. S1c, arrows in a right inset), indicating that they developed into RGCs.

### Suppression of embryoid body (EB) growth by low-dose radiation

**Figure 1:**
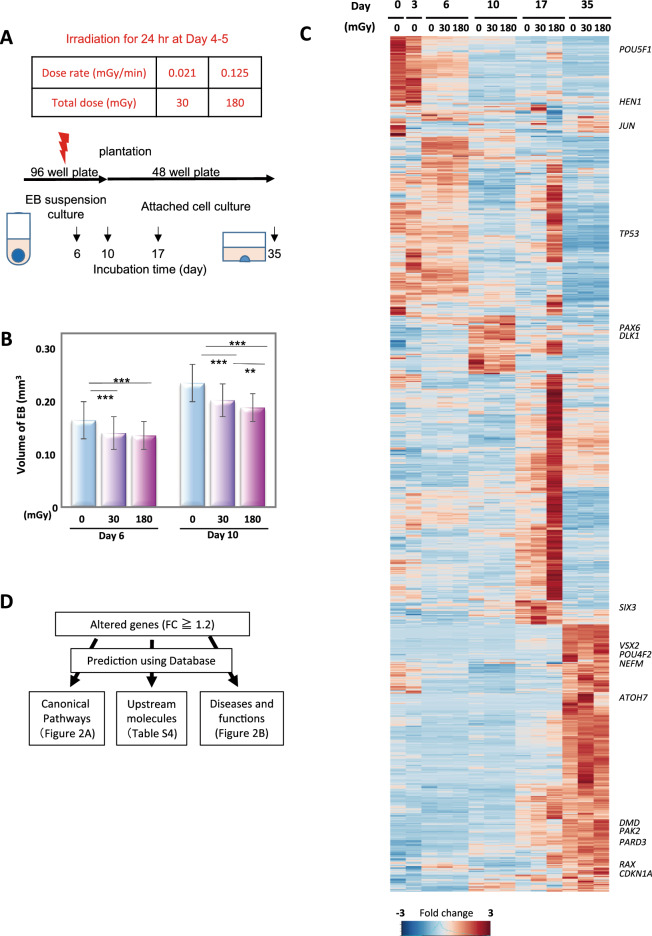
Growth suppression of EB and altered gene expression by low-dose radiation. (**A**) Doses and time course of the exposure experiment. On day 0, iPSCs were placed into 96-well culture plates. After EB formation, the cells were irradiated from days 4 to 5. The doses (dose rates) were 30 mGy (0.021 mGy/min) and 180 mGy (0.125 mGy/min). On day 10, the cells were transferred to 48-well flat bottom culture plates coated with iMatrix-511. On days 0, 3, 6, 10, 17, and 35, RNAs were analysed. (**B**) Growth suppression by low-dose radiation. EB sizes are shown. Data are presented as the mean and standard deviation of 10 EBs. Experiments were repeated three times on different days. ∗∗∗*p* < 0.001, ∗∗*p* < 0.01 (Student’s *t* test). Error bar: average deviation of three experiments. (**C**) Clustering and heatmap of RNA-seq data. RNA including six to twelve EBs at each point was isolated. Of the 26,260 genes, 5,246 genes had > 25 FPKM that met one or more conditions (Supplemental Table S1). Among them, 1,007 genes with changes in expression ≥ 1.4-fold compared with the FPKM for nonirradiated cells on the same day at least once were selected (Supplemental Table S2). After selection, the FPKM of each gene was normalized and clustered. RNA-seq was performed once for days 1, 3, 10, and 17 and three times for days 6 and 35. Each RNA-seq sample included six to twelve EBs. (**D**) IPA schematic. Radiation-induced changes in gene expression were examined by IPA in three independent ways. First, relevant “upstream molecules” were predicted by summarizing the altered genes matched with downstream genes. Second, altered genes were matched to registered genes in each “canonical pathway.” Third, downstream “Diseases and functions” were predicted by matching the altered genes with genes in the database. For all “canonical pathways,” “upstream molecules,” and “diseases and functions,” *P* values and *Z* scores were calculated. The *P* value is the calculated significance using Fisher’s exact test (calculated as − log *p*). The *Z* score is the predicted degree of activation (positive) or inhibition (negative).

The developing cells were exposed to 30 or 180 mGy of irradiation for 24 h from days 4 to 5 and subjected to multiple analyses (Fig. 1A). Irradiation considerably suppressed the growth of embryonic bodies (EBs) (Fig. 1B). On day 6, the volume of EBs decreased from 0.16 ± 0.01 mm^3^ without irradiation to 0.14 ± 0.01 mm^3^ (*p* < 0.001, Student’s *t* test) and 0.13 ± 0.003 mm^3^ (*p* < 0.001) for 30 and 180 mGy, respectively (Fig. 1B). On day 10, the volume decreased from 0.23 ± 0.01 mm^3^ without irradiation to 0.19 ± 0.01 mm^3^ (*p* < 0.001) and 0.18 ± 0.006 mm^3^ (*p* < 0.001) for 30 and 180 mGy (*p* < 0.005), respectively. Irradiation significantly suppressed the growth of EBs to 80% of those of nonirradiated cells on days 6 and 10 in a radiation dose-dependent manner. Although the growth of irradiated EBs was suppressed until day 10, the morphological changes observed with phase-contrast microscopy were similar between irradiated and nonirradiated EBs. Gene expression of cell cycle marker (MKI67) and apoptosis markers showed no change on day 6 (Supple Fig. S2).

### Altered gene expression after low-dose radiation

RNA samples from six to twelve EBs were isolated on days 0, 3, 6, 10, 17, and 35. From days 4 to 5, cells were irradiated with either 30 or 180 mGy. High-throughput RNA sequencing extracted 11,727 genes with > 5 fragments per kilobase of exon per million mapped reads (FPKM) (Supplementary Table S1). Of the 5,426 genes with > 25 FPKM, 1007 genes that changed ≥ 1.4-fold compared with nonirradiated cells on the same day were selected for Fig. 1C (Supple Table S2). These results indicated that two doses of irradiation caused different gene induction profiles in the developing cells.

**Figure 2:**
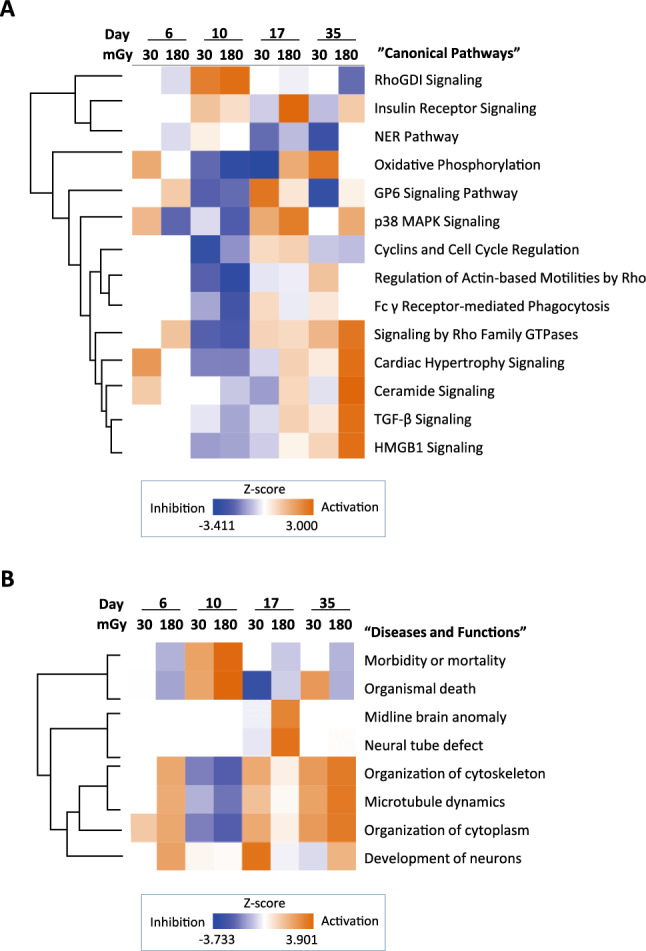
Pathways altered by low-dose radiation. (**A**) Clustering heatmap of altered “canonical pathways”. IPA analysis revealed 212 canonical pathways that differed by more than 2–log (*p* value) in expression in irradiated cells compared to nonirradiated cells according to the RNA-seq data. Among them, 13 with high *Z* scores (cut-off, 2.5) were clustered. (**B**) Clustering heatmap of altered “Diseases and functions.” Comparison between the effects of radiation doses (0, 30, or 180 mGy) on days 6, 10, 17, and 35. The IPA-calculated *p* value and *Z* score for each “functions and diseases” pathway differed by > 2 of –log (*p* value) in RNA-seq. Clustering of the eight pathways that had high *Z* scores (cut-off, 3.0) is shown.

Using transcriptome data, knowledge-based pathway analysis (Ingenuity Pathway Analysis [IPA], QIAGEN, Dusseldorf, Germany) was performed in three categories (Fig. 1D). They were “Canonical Pathways” (Fig. 2A, Supple Table S3), “Upstream Regulators” (Supple Table S4), and “Diseases and functions.” (Fig. 2B). For the analysis, the likelihoods (*p* values) and the activation/inhibition scores (*Z* scores) of biological pathways were calculated using originally designed formulas^20^.

### Altered Rho signaling after low-dose radiation

Fourteen canonical pathways had *Z* scores > 2.5 at least one time/dose point (Fig. 2A, Supple Table S5). Among them, some Rho GTPase-related signals were included. Rho GTPases play important roles in neural development through cell proliferation, apoptosis, migration, cytoskeletal reorganization, and membrane trafficking^21^. “RhoGDI signaling,” which has opposite effects to Rho GTPases^22^, was activated on day 10 (Supple Fig. S3a) and inactivated on day 35 by irradiation (Fig. 2A).

In contrast, “Signalling by Rho Family GTPases” was inactivated on day 10 but activated on day 35 (Fig. 2A, Supple Fig. S3b). “Regulation of Actin-based Motilities by Rho” was also altered. Low-dose radiation inactivated Rho signalling just after exposure and activated it later in development (Fig. 2A).

Biological prediction was performed based on the fold changes of the identified upstream molecules in IPA. Dexamethasone and several hormones were predicted. TP53, RAS, MYC, and other transcription factors (TFs) were also proposed (Supplementary Table S4).

### Neural development was altered by 30 and 180 mGy of irradiation

Altered phenotypes were predicted with IPA. All eight identified radiation response categories for diseases and functions with Z scores > 3.0 were related to neural development (Fig. 2B). “Morbidity or Mortality” and “Organismal Death” were activated dose-dependently on day 10, suggesting that the irradiated cells began to die on day 10. “Midline Brain Anomaly” and “Neural Tube Defect” clusters were activated with 180 mGy of radiation on day 17. The clusters of “Organization of Cytoskeleton”, “Microtubule Dynamics,” and “Organization of Cytoplasm” were significant for neural development. They were activated by irradiation on days 17 and 35.

We focused on the cluster “Development of Neurons” on day 35 (Fig. 2B), where 30 mGy of radiation inactivated the “Development of neurons” cluster (*Z* =  − 0.403, − log *p* = 9.011) (Supple Fig. S4), but 180 mGy activated it (*Z* = 2.065, − log *p* = 14.959) (Supple Fig. S5) (Supple Table S6; 1344 genes registered under “Development of Neuron” in IPA, altered genes on day 35). The expression levels of 95 and 104 genes were modified by 30 mGy and 180 mGy of irradiation on day 35, respectively. On day 35, the levels of vimentin (*VIM*), and Par-3 family cell polarity regulator (*PARD3*) were enhanced by 180 mGy (Supple Figs. S5, S6d, Fig. 4D). They are members of the Rho family of GTPases (Supple Fig. S3b) and play transcriptional roles in cell proliferation and stress responses^23–25^.

### Different effects on retinal development after 30 and 180 mGy of irradiation

**Figure 3:**
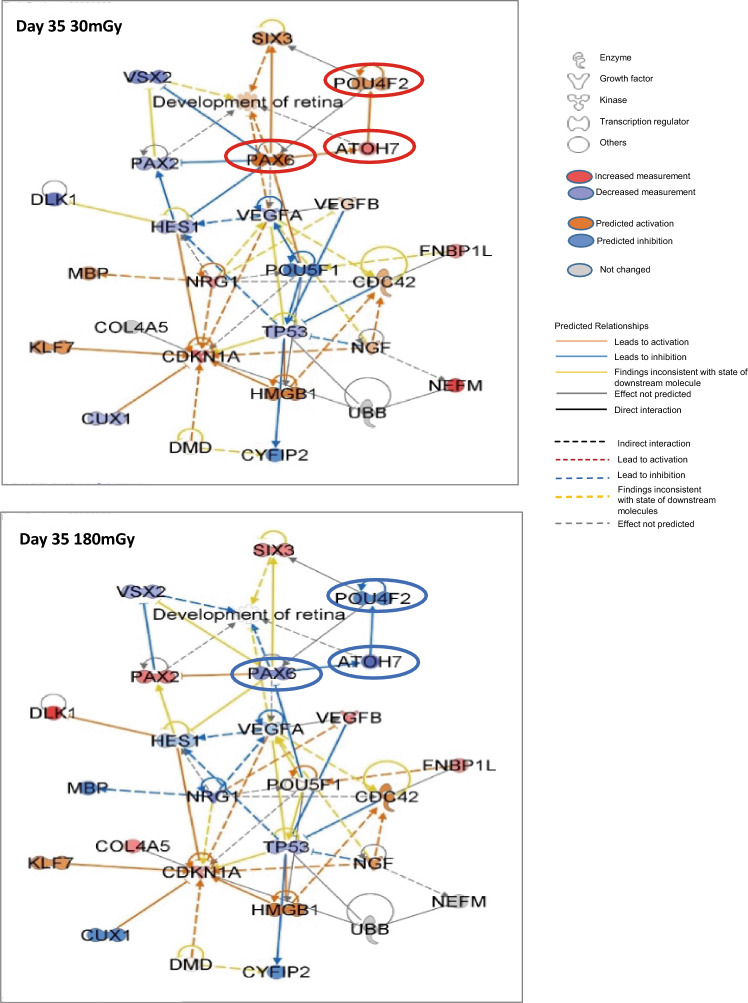
Genes associated with the “Development of the retina” were differentially altered by 30 mGy and 180 mGy. Network of genes altered as determined from the list of altered genes in “Development of retina” (Supplemental Table S8) and other genes selected on day 35. Genes with fold changes > 1.2 were increased (red), and those <  − 1.2 were decreased (purple). Predicted activation (orange) and predicted inactivation (blue) of genes without their own activation/inactivation were calculated by IPA with altered expression of related genes. Genes with predicted relationships are connected by lines. Top: 30 mGy. Left bottom. Bottom: 180 mGy. Each dose sample for single RNA-seq included 6 to 12 EBs. RNA-seq was repeated three times for each dose.

Based on the fold changes of FPKM in RNA-seq, 26 genes were extracted from 129 retinal development-related genes (Supple Tables S7, S8), and retinal development diagrams were generated using IPA (Fig. 3). In RGC development, the transcriptional axis consisting of PAX6, ATOH7, and POU4F2 is well recognized, and they functioned inversely after irradiation of 30 and 180 mGy on day 35. While there was a tendency that “retinal development” was facilitated by 30 mGy (Fig. 3, top) and suppressed by 180 mGy (Fig. 3, bottom), they were not statistically significant.

**Figure 4:**
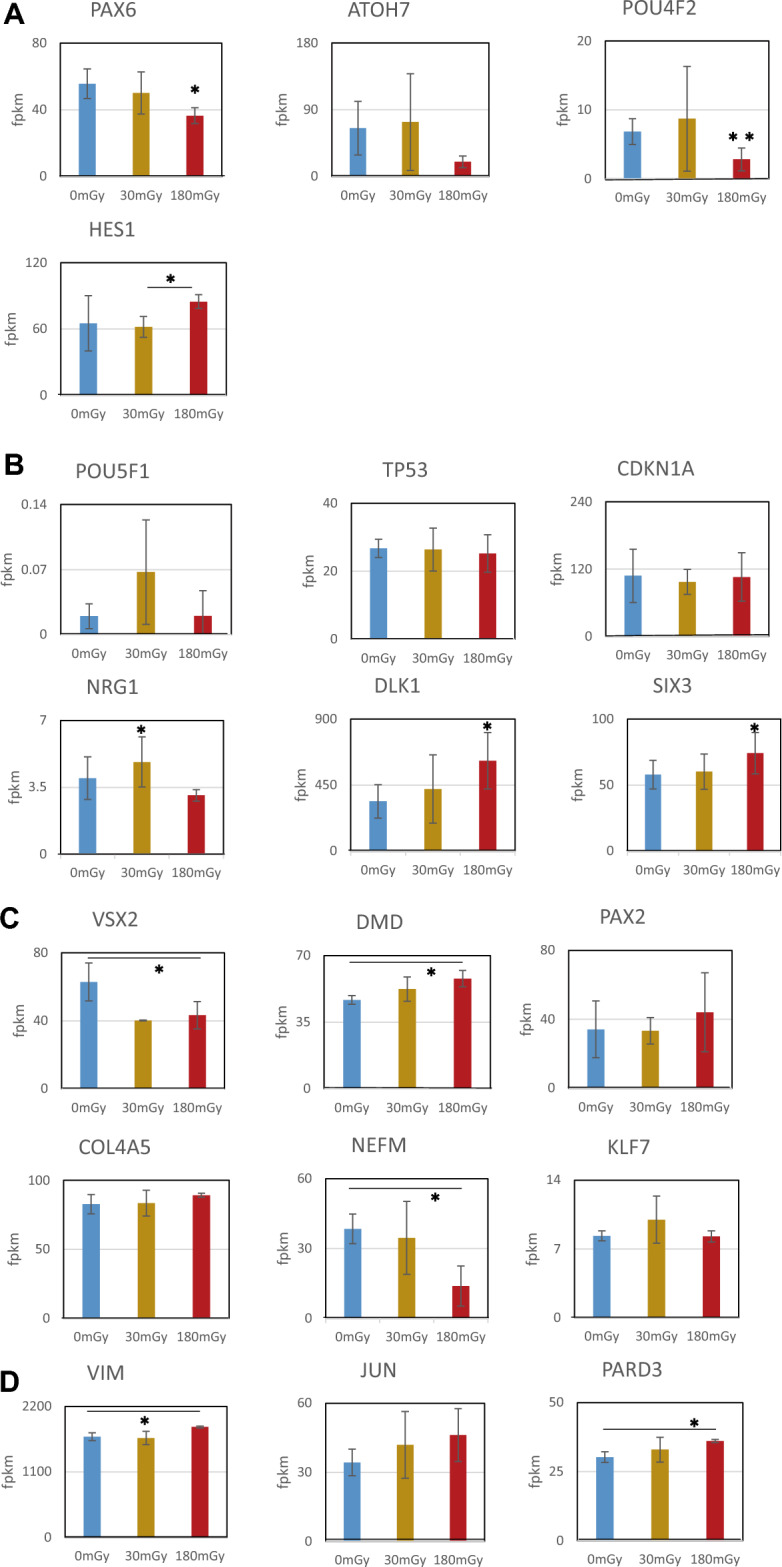
Expression of retinal and neural development-related genes on Day 35. Cells were exposed to 0, 30, or 180 mGy of radiation from days 4 to 5 of development. RNA was extracted on day 35. Each sample includes 6 to 12 EBs. (**A**) Main four TFs for RGC development. Altered expression of *PAX6, ATOH7, POU4F2,* and *HES1* by radiation was found. (**B**) Early-stage retinal development-related genes. Altered expression of *POU5F1, TP53, CDKN1A*, *NRG1*, *NEFM*, *DLK1*, and *SIX3* was found. (**C**) Late-stage retinal development-related genes. Altered time-course expression of *VSX2*, *DMD*, *PAX2, COL4A5*, *NEFM*, and *KLF7* was found. (**D**) Neuronal development-related genes. *VIM*, *JUN* and *PARD3* are members of the Rho family of GTPases and are involved in neuronal development. After 180 mGy of irradiation, their expression was upregulated on day 35. The mean FPKM and average of the absolute deviations detected by RNA-seq three times are shown (n = 3). Error bar: average absolute deviation, *p < 0.05, **p < 0.01 (Student’s paired* t* test).

The dose effects of the calculated gene expression levels in retinal development using triplicated RNA-seq on day 35 are shown in Fig. 4. We classified the genes of “Development of retina” into three groups (Fig. 4A–C). Figure 4A shows the expression of *PAX6*, Hes family bHLH transcription factor 1 (*HES1*), *ATOH7*, and *POU4F2*. They are the main essential TFs for RGC development^17, 18, 26^.

**Figure 5:**
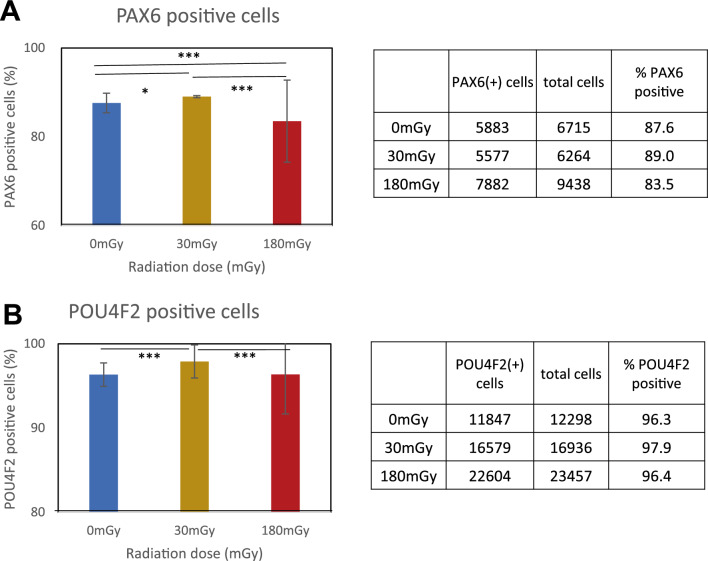
Immunofluorescence of PAX6 and POU4F2 on day 35 after 30 or 180 mGy of irradiation from day 4 to 5. Immunofluorescence with anti-PAX6 or anti-POU4F2 antibody and beta-tubulin were stained. DAPI was analyzed with IN CELL Analyzer. Nuclear area was identified with fluorescence intensity and area size of DAPI. Then, fluorescence intensity of the target in the nuclear area was analyzed. (**A**) PAX6-positive cells. (**B**) POU4F2-positive cells. *P < 0.05, ***P < 0.001 (chi-squared test) (n = 3). Experiment was repeated three times and representative data was presented.

In the time course of gene expression investigated using RNA-seq (Suppl. Fig. S6a), *PAX6* expression levels increased from day 6 and peaked on day 10, and the peak was dose-dependently facilitated by irradiation (Suppl. Fig. S6a). Alternatively, *ATOH7* and *POU4F2* were not expressed in the early days. HES1 antagonizes PAX6 to suppress the expression of ATOH7^27, 28^. On day 35, the expression levels of *PAX6* and downstream *POU4F2* significantly decreased after 180 mGy of radiation (*PAX6*: *p* < 0.05. *POU4F2*: *P* < 0.01, Student’s paired *t*-test). Simultaneously, expression of *HES1* increased (*p* < 0.05). Next, we checked the number of PAX6- or POU4F2-positive cells on day 35 (Fig. 5). PAX6-positive cells were 87.6%, 89.0%, and 83.5% after 0 mGy, 30 mGy, and 180 mGy, respectively. They significantly (0 mGy vs 30 mGy: *p* = 0.013, 0 mGy vs 180 mGy: *P* < 0.001, 30 mGy vs 180 mGy: *P* < 0.001, Chi-squared Statistic). POU4F2-positive cells were 96.3%, 97.9%, and 96.6%, respectively (0 mGy vs 30 mGy: *P* < 0.001, 0 mGy vs 180 mGy: *P* = 0.91, 30 mGy vs 180 mGy: *P* < 0.001, Chi-squared test).

The other genes were sorted according to the time course of their expression profiles. Figure 4B shows the expression of six genes on day 35 that had early peaks in their time-course expression profiles (Suppl Fig. S6b), including *POU5F1*, tumor protein p53 (*TP53*), cyclin-dependent kinase inhibitor 1A (*CDKN1A*), neuregulin1 (*NRG1*), delta-like noncanonical Notch ligand 1 (*DLK1*), and SIX homeobox 3 (*SIX3*). In the time course (Suppl Fig. S6b), *POU5F1* levels decreased on day 10. On day 35, 30 mGy of irradiation facilitated *NRG1*(*p* < 0.05). In contrast, the gene expression of *DLK1* and *SIX 3* was upregulated by 180 mGy (*p* < 0.05). They are essential in retinal vascular morphogenesis^29, 30^ and optic nerve formation^31, 32^, respectively. The expression levels of these genes were increased during early development, and they decreased by day 35. However, after exposure of 180 mGy, those of *DLK1* and *SIX 3* did not decreased enough on day 35 (Fig. 4B, Suppl Fig. S6b).

Figure 4C shows retinal development-related genes with expression peaks in the late days, including visual system homeobox 2 (*VSX2*), dystrophin (*DMD*), paired box 2 (*PAX2*), collagen type IV alpha 5 chain (*COL4A5*), neurofilament medium chain (*NEFM*), KLF transcription factor 7 (*KLF7*). VSX2 is a crucial player in eye and retinal development^33^. DMD is essential for visual functional development^34, 35^. PAX2 and COL4A5 are required for optic vesicle formation^36, 37^ and retinal layer formation^38^, respectively. NEFM encodes a component of the neurofilament and is upregulated in damaged neurons^39^. KLF7 is expressed during RGC differentiation and plays an important role in cell maturation^40^. On day 35, while 180 mGy of irradiation suppressed the expression of *VSX2* (*p* < 0.05) and *NEFM* (*p* < 0.05), it upregulated *DMD* expression (*p* < 0.05) (Fig. 4C).

Figure 4D shows the expression of *VIM, JUN,* and *PARD3*. *VIM* and *PARD3* were enriched in “Development of Neurons” on day 35 after 180 mGy of irradiation significantly (*p* < 0.05) (Fig. 4D, Supple Fig. S6d). Up-regulation of *VIM* and *PARD3* suggested glial cell proliferation^41^ and asymmetric division of glial cells^42^. To confirm the effects of low-dose radiation on cellular proliferation on day 35, cell cycle analysis was performed. There were few effects of exposure on the cell cycle distribution on day 35 between nonirradiated and irradiated cells (Supple Fig. S7).

## Discussion

**Figure 6:**
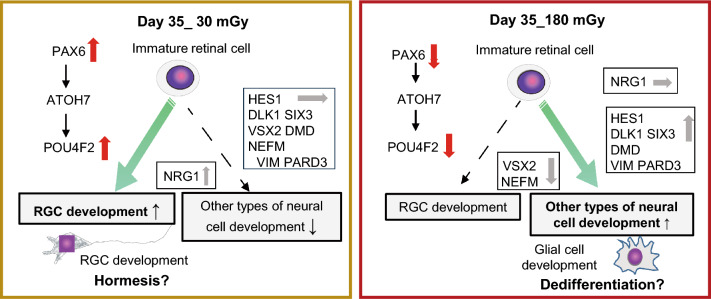
Hypothesis of unstable gene expression and fluctuating fate determination. Low-dose radiation induces unstable gene expression and alters fate determination in RGCs.retinal development. *Left*: Radiation with 30 mGy results in an increased percentage of POU4F2-positive cells on day 35*,* suggesting hormesis effects. *Right*: Radiation with 180 mGy results in the downregulated expression of *PAX6* and its downstream *POU4F2* on day 35. Rather than RGC development, neural development, suggesting the dedifferentiation and reproduction of other types of cells, such as glial cells. Overall, low-dose radiation causes unstable gene expression that modifies fate determination of RGCs.

In this study, we examined the effects of 30 and 180 mGy of low-dose radiation on developing human retinal progenitor cells. Genome-wide gene expression analysis revealed the possibility that the two low doses of ionizing radiation induced different effects, hormesis and dedifferentiation, in developing retinal cells (Fig. 6).

We focused on the activation of retinal development after 30 mGy of exposure on day 35 (Fig. 3). Even though the upregulated expression of *PAX6* and *POU4F2* was not statistically significant, immunofluorescence revealed increases in the proportion of PAX6- and POU4F2-positive cells after 30 mGy (Fig. 5). We suppose that the phenomenon is hormesis, in which the differentiation of RGCs is stimulated (Fig. 6, left). Nevertheless, gene expression after 30 mGy showed a large fluctuation range (Fig. 4A). Therefore, we speculate that there are further regulatory mechanisms and there is branch point around the dose.

Previously, we reported the effects of low-dose radiation on neural progenitor cells^9^. In that report, the examination was limited to just after exposure. A total of 31 mGy of exposure affected several pathways related to the interferon pathway, cell junctions, myogenesis, and insulin-like growth factor (IGF). The effects of 31 mGy were minimal, compared to those of more than 100 mGy^9^. In this study, we determined the gene expression of retinal progenitor cells for a month after exposure. TP53 is one of the most important genes in irradiated cells^9^ and neural development^43^. CDKN1A is downstream of TP53 and prevents neural cell death as a player in the cell cycle checkpoint^44^ and regulates neurite remodeling through Rho-kinase^45^. TP53 and CDKN1A showed little change of gene expression after the exposure (Fig. 4B). Instead, we revealed other altered expression of genes and signallings in the retinal progenitor cells for a month after exposure. On day 10, “RhoGDI signalling”, which is a negative regulator of Rho-GTP signalling, and “insulin receptor signalling” were activated (Fig. 2A). “p38 MAPK Signalling” was activated later on day 17 (Fig. 2A). This pathway is activated in various cells by low-dose ionizing radiation and ultraviolet radiation^46^. “Insulin Receptor Signalling,” “Oxidative Phosphorylation,” and some Rho-related signalling were also inhibited and activated (Fig. 2A, Supplemental Fig. S3). These pathways are related to inflammation and neural/retinal development^46–49^. Neural development consists of neurogenesis, migration, differentiation, and outgrowth^49^. *PAX6* and *NGR1* play a role in the early stages of RGC development, such as neurogenesis and migration (Supplemental Fig. S6a, b). NRG1 plays a critical role in the growth and development of multiple organ systems and is present in adult human RGCs^50^. A *NRG1* homolog was reported to participate in the axon growth of rats through Rho signaling^51^ and in the regeneration of zebrafish^52^. Therefore, we can regard that the increased expression of *NRG1* participated the hormesis effect after the 30 mGy of exposure. While < 100 mGy of irradiation has been reported to evoke hormesis, the molecular mechanism is still unclear^53^. In this study, we revealed the alteration of Rho signaling and related genes involved in the hormesis effects.

We found that 180 mGy of exposure conversely affected “Development of neuron” pathways to “Retinal development” (Fig. 3, Supplemental Fig. S5). Among many activated genes associated with the “development of neurons”, VIM and PARD3 are expressed in neural progenitor cells^23–25^, and we speculate that their upregulated expression indicates the dedifferentiation and regeneration of glial cells (Fig. 4D). The increased *VIM* levels suggest an increase in glial cell number^41^. Furthermore, the increased PARD3 levels regulate the asymmetrical division of glial cells to determine the fate of daughter cells^42^. Since POU4F2 has a role in suppressing the differentiation of retinal cell types other than RGCs^19^, the decrease in *POU4F2* by 180 mGy also supports the dedifferentiation and reproduction of other cells (Fig. 4A). In this experiment, 180 mGy of irradiation prolonged the expression of *DLK1* and *SIX3* until day 35(Suppl Fig. S6b). The peak expression levels of *DLK1* and *SIX3* were on days 10 and 17, respectively, and they decreased on day 35. However, 180 mGy of exposure inhibited the decrease (Suppl Fig. S6b)*.* As the expression of *DLK1* is increased during hepatic cell regeneration^54^, we speculate that retinal cell regeneration occurred at this time point. DMD plays multiple roles in eye development^35^. In this study, the expression of *DMD* was also upregulated after 180 mGy of irradiation on day 35. Since mutation in DMD is reported to cause retinal weakness in response to ischemia^55^, the upregulation of *DMD* suggests activation of retinal stress response. On the other hands, the expression of *NEFM* was downregulated after 180 mGy of irradiation on day 35. As NEFM increases in damaged neuron, why it decreased after exposure to 180 mGy is unclear. We expect that the findings may contain important information for understanding the functions of NEFM. Furthermore, exposure to 180 mGy activated “Signaling by Rho family GTPases,” “Ceramide signaling,” and “TGF-β signaling” (Fig. 2A). These pathways play important roles in cellular adhesion, movement, and transcriptional regulation in neural development. Together, 180 mGy of low-dose irradiation caused fluctuations in gene expression, which interfered with the actual timing of retinal development and led dedifferentiation and regeneration of developing retinal cells.

The increased risk of NTG by irradiation has been reported^6, 56^, although its mechanism remains unclear. In the hippocampus of adult brains, radiosensitive neural precursor cells exist, and radiation exposure can trigger the development of dementia several years later^3, 11–13^. There is a possibility that the same processes occur in the hippocampus are also occurring in the retina. Dementia arising from the irradiated neural precursor cells in the hippocampus and NTG arising from retinal progenitor cells could follow the same paradigm. Whether retinal precursor cells exist in the adult human retina is still under debate. Since there is still no established treatment for degenerative retinal and optic nerve diseases, including glaucoma, understanding the mechanisms of RGC differentiation and the stress response of retinal stem cells is necessary. In this study, several pathways showed opposite reactions to 30 mGy and 180 mGy in the early days after exposure. These pathways have become important switches for the subsequent development of retinal organoids.

In summary, we demonstrated unstable gene expression in the developing human retina after low-dose irradiation for a long period of 1 month. Rho signalling is one of the most important pathways influencing the effects of low-dose radiation on human retinal development. The altered gene expression induced by two different low-dose irradiations caused fluctuating fate determination in retinal organoid development.

## Materials and methods

### Ethics statement

All experiments were performed according to the guidelines for biological research of the University of Tokyo. Data from human iPSCs were handled in accordance with the Department of NBDC Program (hereinafter, NBDC) of the Japan Science and Technology Agency (JST). Radiation exposure experiments were performed in accordance with the Regulation on Prevention of Ionizing Radiation Hazards (Ministry of Labour Order No. 41 of September 30, 1972).

### Cell culture of RGCs from iPSCs

We generated a feeder-free culture system of iPSCs differentiated into RGCs to avoid possible contamination from feeder cells. iPSCs derived from healthy humans (201B7) were supplied by Kyoto University through RIKEN BRC (Tsukuba, Japan)^57^, and cells were maintained in mTeSR1 medium (STEMCELL Technologies, Vancouver, BC). After culture for 2–3 weeks, subconfluent cells were treated with 10 µM ROCK inhibitor Y-27632 for 1 h, washed once with PBS (−) and treated with Accutase (Nacalai Tesque Inc., Kyoto, Japan) for 5 min at 37 °C. For EB formation, the single cell suspension was transferred into 96-well polystyrene round-bottom plates (SUMITOMO Bakerite, Tokyo, Japan) at a concentration of 9000 cells per well in mTeSR1 supplemented with 10-μM Y-27632 and 10-ng/mL Noggin (Fujifilm Wako Pure Chemical, Osaka, Japan) and cultured at 37 °C with 5% CO_2_ (day 0). On day 3, the culture medium was substituted for DMEM/F12 (Sigma) containing 20% KnockOut Serum Replacement (Thermo Fisher Scientific, Waltham, MA), 0.1-mM, 2-mercaptoethanol (Thermo Fisher Scientific), 1× GlutaMAX (Thermo Fisher Scientific), 1× nonessential amino acids (NEAA) (Fujifilm Wako Pure Chemical), 100 ng/mL of Noggin, 100 units/mL of penicillin and 100 μg/mL of streptomycin. The culture medium was changed three times per week. The culture plates were kept in a CO_2_ incubator. A Cs137 gamma-ray source was placed near the incubator for 24 h on days 4 to 5 (Fig. 1A). After 10 days, the EBs were cultured in a 48-well flat bottom culture dish coated with iMatrix-511 (2.4 μg/mL in PBS) (Fujifilm Wako Pure Chemical) in DMEM-F12 with 2% B-27 (Thermo Fisher Scientific), 1% N-2 (Fujifilm Wako Pure Chemical), 1× GlutaMAX, 1× NEAA, 10-μM DAPT (Fujifilm Wako Pure Chemical), 125-ng/mL iMatrix-511 and 10-ng/mL DKK-1 (Pepro Tech Inc., Cranbury, NJ) thrice per week until day 35.

We observed time-dependent morphological changes in the hiPSC-derived embryonal body using phase-contrast microscopy (Supplemental Fig. S1a). These morphological changes were similar to those previously reported^14, 15^.

### Monitoring of RGC development using qPCR

To confirm the observed developmental changes, we evaluated the expression of several eye- and retina-specific marker genes (Supplemental Fig. S1b). Retinal and anterior neural fold homeobox (*RAX*) are expressed early in the eye primordia and are essential for retinal development^16^. *PAX6* is essential for eye development and a candidate gene for congenital aniridia^17^. *POU4F2/BRN3B* is expressed in RGCs^18^ and suppresses the differentiation of other types of retinal cells. Total RNA was isolated from three to six EBs on days 3, 10, 17, 25, and 35 using an RNeasy microRNA isolation kit (QIAGEN, Japan). Isolated RNAs were treated with reverse transcriptase (TaKaRa, Ohtsu, Japan) to generate cDNA. The expression of marker genes of eyes and retinal development (*RAX*, *PAX6,* and *POU4F2*) was assessed using real-time qPCR with the following primers:

- *RAX* forwards: CCCTAAGCGTGCTTTCAGGA
- *RAX* reverse: TTTGCTCAGGACCGACAGAC
- *PAX6* forwards: TGGCTCACCAAGGCGAAATA
- *PAX6* reverse: CCGAGCAGTTGAGTCATTCAG
- *POU4F2* forwards: GAACGAGCGAACAACTGAGC
- *POU4F2* reverse: CTCTGAAGAAGCCGAGGTGG
- *SCS* (18S) forwards: TGTGTGAAAGATTAAGCATGCA
- *SCS* (18S) reverse: GCGACCAAAGGAACCATAACTG

We used Premix Ex Taq (Perfect Real-Time, Takara bio) and a CFX Connect Real-Time PCR Detection System (Bio-Rad, Japan). The results were normalized to *SCS* (18S) expression. All experiments were run in triplicate (Supplemental Fig. S1b).

### Immunofluorescence

The attached cells were fixed with 10% paraformaldehyde in PBS for 10 min at room temperature, washed three times with PBS, and permeabilized with 0.5% Triton-X and 0.1% SDS in PBS for 5 min. After incubation in blocking buffer (10% horse serum in PBS) for 30 min, the cells were incubated with primary antibodies diluted in 1% bovine serum albumin in PBS for 2 h at room temperature. Anti-neuron-specific beta-tubulin III (MAB1195) antibodies were purchased from R&D Systems (Minneapolis, MN). The anti-Pax6 antibody (Cat. No. 901301) was purchased from BioLegend, and the anti-BRN3B/POU4F2 (sc-31989) antibody was purchased from Santa Cruz Biotechnology (Santa Cruz, CA, USA). A series of Alexa Fluor-conjugated antibodies (Thermo Fisher Scientific) were used as secondary antibodies. Chromatin was stained with DAPI (4′,6-diamidino-2-phenylindole) (Merck-Millipore, Burlington, MA, USA) and observed using a fluorescence microscope (Leica Microsystems, Tokyo, Japan) (Supple Fig. S1c). Analysis of stained cells was performed with an IN Cell Analyzer 6000 (GE Health Care, Tokyo, Japan) (Fig. 5). Cell cycle analysis of DAPI-stained cells was performed using MetaMorph 7.10.5 software and Cell Cycle Application Module (Molecular Devices, Tokyo, Japan) (Supplemental Fig. S7). Experiments were repeated three times.

### Irradiation with γ-rays

Sealed radioactive Cs-137 of 18.5 GBq was used for irradiation with γ-rays. The cells were placed in a CO_2_ incubator next to the radiation source. The radiation dose was measured using a Microstar dosimeter (Nagase Landauer, ltd., Tsukuba, Japan). The total absorbed dose was either 30 or 180 mGy, calculated from radiation rates at either 0.021 or 0.125 mGy/min, respectively. The cells were irradiated for 24 h from days 4 to 5 of differentiation (Fig. 1A).

### Measurement of EB size

We measured the diameter and calculated the volume of EBs with photographs taken by a phase-contrast microscope on days 6 and 10. Ten EBs were analysed per experiment (N = 10). The experiments were repeated three times. Representative data are shown in Fig. 1B. The statistical significance was calculated with Student’s *t*-test.

### Gene expression analysis via RNA-seq

On days 0, 3, 6, 10, 17, and 35, six to twelve EBs per experimental condition were pooled into a tube. Total RNA was isolated using an RNeasy microRNA isolation kit (QIAGEN, Japan). Isolated RNA was treated with reverse transcriptase (TaKaRa, Ohtsu, Japan) to generate cDNA and analysed using a next-generation sequencer, HiSeq 2000/2500 or MiSeq (Illumina, San Diego, CA, USA). Reads were mapped to the hg19 genome by Hisat2^58^. Gene expression was quantified using Cufflinks^59, 60^ with the hg19 refseq table used as a reference. The data were visualized using the Integrative Genomics Viewer (http://software.broadinstitute.org/software/igv/) using the hg19 human genome database. RNA-seq was performed once for days 1, 3, 10, and 17 and three times for days 6 and 35. Each RNA-seq sample included six to twelve EBs.

### Gene ontology analysis using IPA

The data were analysed using IPA software (QIAGEN Bioinformatics, CA, US, https://www.qiagenbioinformatics.com/products/ingenuity-pathway-analysis). The cutoff was set at < 1.2-fold change. The *p* values were determined using Fisher’s exact test (calculated as − log *p*). The Z score is a statistical measure of how closely the actual expression pattern of molecules in the dataset compares to that expected based on the literature for a particular annotation^20^. A positive score indicates the degree of predicted activation of the expected network, and a negative score indicates predicted inactivation.

### Statistical analysis

Statistical analyses for all experiments are described in the figure legends, the method details, and summarized in Supplemental Table S9. Briefly, RNA-seq for day 35 was repeated three times, but on other days (days 0, 3, 6, 10, 17), it was performed once with technical duplicates. Every RNA-seq sample included 6 to 12 EBs (Figs. 1C, 2, 3). The experiments of EB size (n = 10) were repeated three times. They were analyzed with Student’s *t*-test (Fig. 1B). Gene expression on day 35 were triplicated. Average of the three fpkm were analyzed with Student’s *t*-test (Fig. 4). In the analysis of immunofluorescence was repeated three times and the proportion numbers of the positive- cells were analyzed with Chi square test (Fig. 5). 

### Supplementary Information

Supplementary Tables.

Supplementary Figures.

## Data Availability

Data are available in the Gene Expression Omnibus datasets. The RNA-seq dataset is available under accession number GSE142658.
