# Supplementary material for: Low-dose radiation induces unstable gene expression in developing human iPSC-derived retinal ganglion organoids
Source: Sci Rep. 2023 Aug 9;13:12888. doi: 10.1038/s41598-023-40051-6 (PMC10412642; doi:10.1038/s41598-023-40051-6)
Supplement: Supplementary file 2 — Supplementary Figures. [file 41598_2023_40051_MOESM2_ESM.pptx]

## Slide 1
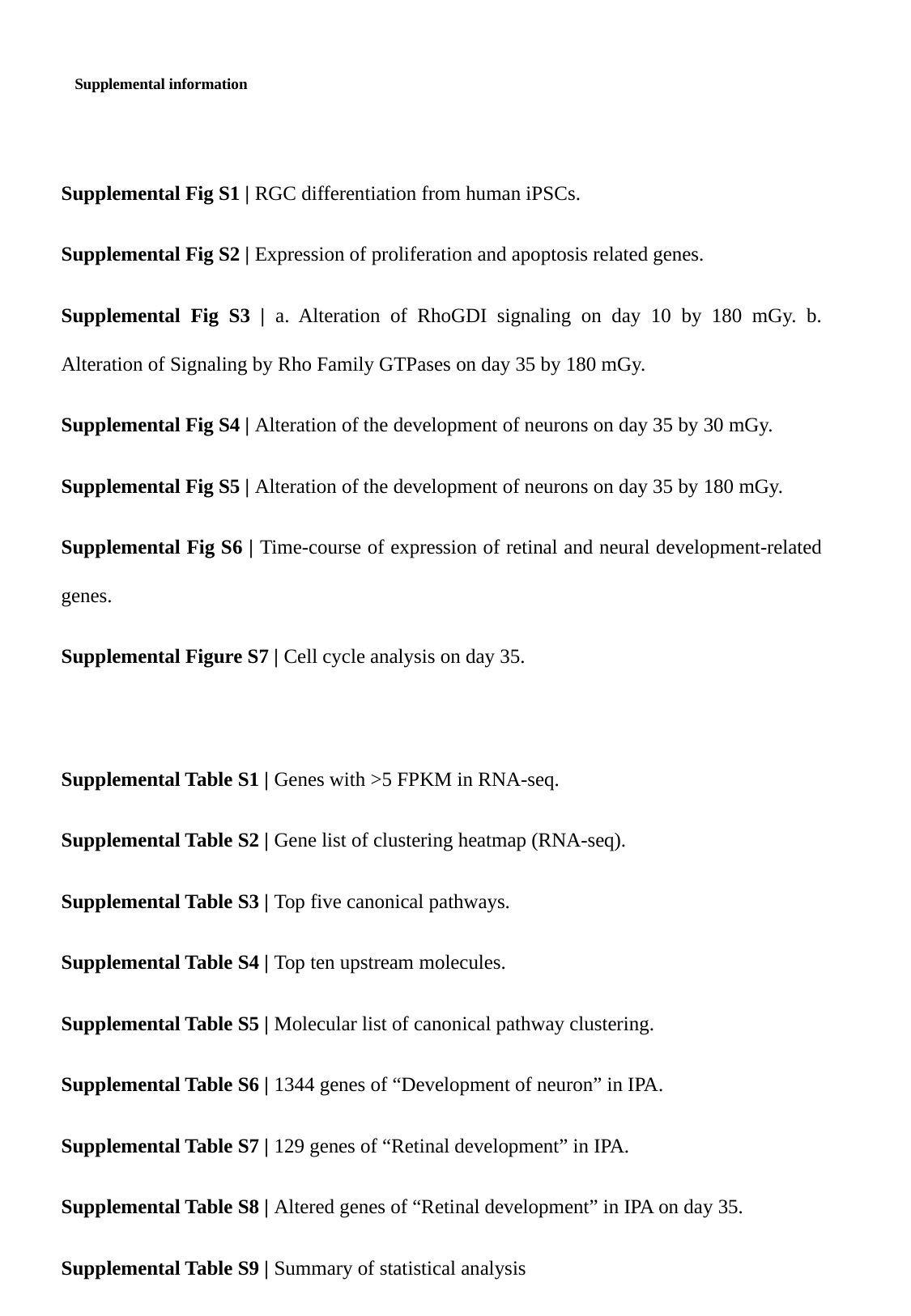

# Supplemental information
Supplemental Fig S1 | RGC differentiation from human iPSCs.
Supplemental Fig S2 | Expression of proliferation and apoptosis related genes.
Supplemental Fig S3 | a. Alteration of RhoGDI signaling on day 10 by 180 mGy. b. Alteration of Signaling by Rho Family GTPases on day 35 by 180 mGy.
Supplemental Fig S4 | Alteration of the development of neurons on day 35 by 30 mGy.
Supplemental Fig S5 | Alteration of the development of neurons on day 35 by 180 mGy.
Supplemental Fig S6 | Time-course of expression of retinal and neural development-related genes.
Supplemental Figure S7 | Cell cycle analysis on day 35.
Supplemental Table S1 | Genes with >5 FPKM in RNA-seq.
Supplemental Table S2 | Gene list of clustering heatmap (RNA-seq).
Supplemental Table S3 | Top five canonical pathways.
Supplemental Table S4 | Top ten upstream molecules.
Supplemental Table S5 | Molecular list of canonical pathway clustering.
Supplemental Table S6 | 1344 genes of “Development of neuron” in IPA.
Supplemental Table S7 | 129 genes of “Retinal development” in IPA.
Supplemental Table S8 | Altered genes of “Retinal development” in IPA on day 35.
Supplemental Table S9 | Summary of statistical analysis

## Slide 2
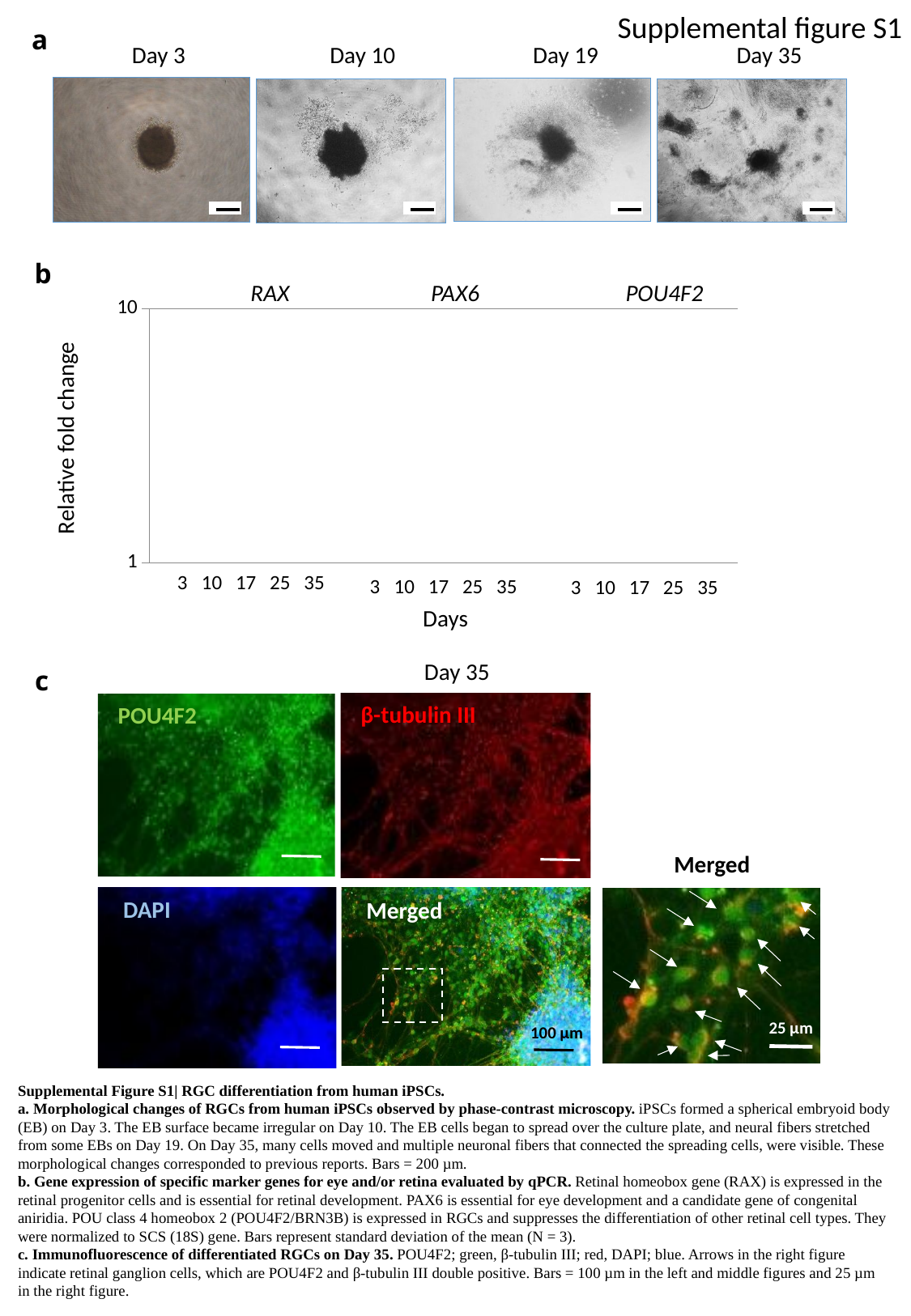

Supplemental figure S1
a
Day 3
Day 10
Day 19
Day 35
b
RAX
PAX6
POU4F2
### Chart
| Category | | | | | |
|---|---|---|---|---|---|
| RX | 1.0 | 62.852434162247185 | 3907.5437404505406 | 2234.2016835435493 | 807.5068571928917 |
| Pax6 | 1.0 | 1.8561256113264097 | 23.567408378435903 | 35.5928372993007 | 28.541283487637475 |
| Brn3b | 1.0 | 2.1010906259493494 | 0.8030762910733821 | 5.367525220402378 | 2636.4492868934317 |Relative fold change
3
10
17
25
35
3
10
17
25
35
3
10
17
25
35
Days
Day 35
β-tubulin III
POU4F2
Merged
DAPI
Merged
25 μm
100 μm
c
Supplemental Figure S1| RGC differentiation from human iPSCs.
a. Morphological changes of RGCs from human iPSCs observed by phase-contrast microscopy. iPSCs formed a spherical embryoid body (EB) on Day 3. The EB surface became irregular on Day 10. The EB cells began to spread over the culture plate, and neural fibers stretched from some EBs on Day 19. On Day 35, many cells moved and multiple neuronal fibers that connected the spreading cells, were visible. These morphological changes corresponded to previous reports. Bars = 200 µm.
b. Gene expression of specific marker genes for eye and/or retina evaluated by qPCR. Retinal homeobox gene (RAX) is expressed in the retinal progenitor cells and is essential for retinal development. PAX6 is essential for eye development and a candidate gene of congenital aniridia. POU class 4 homeobox 2 (POU4F2/BRN3B) is expressed in RGCs and suppresses the differentiation of other retinal cell types. They were normalized to SCS (18S) gene. Bars represent standard deviation of the mean (N = 3).
c. Immunofluorescence of differentiated RGCs on Day 35. POU4F2; green, β-tubulin III; red, DAPI; blue. Arrows in the right figure indicate retinal ganglion cells, which are POU4F2 and β-tubulin III double positive. Bars = 100 µm in the left and middle figures and 25 µm in the right figure.

## Slide 3
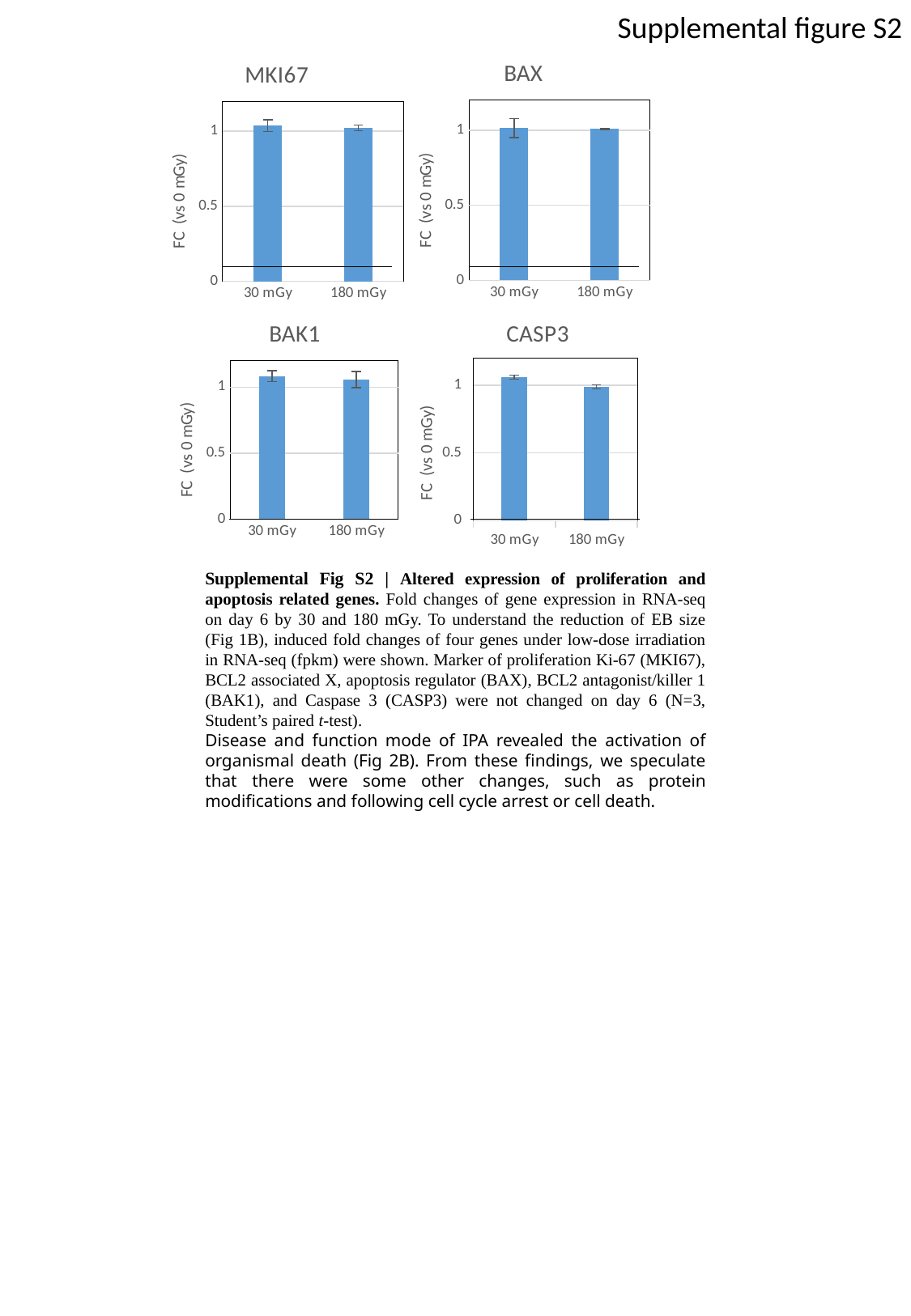

Supplemental figure S2
### Chart: BAX
| Category | Day 6 |
|---|---|
| 30 mGy | 1.0139980701987539 |
| 180 mGy | 1.0073512754053335 |
### Chart: MKI67
| Category | Day 6 |
|---|---|
| 30 mGy | 1.0371621308848744 |
| 180 mGy | 1.021893401751254 |
### Chart: CASP3
| Category | Day 6 |
|---|---|
| 30 mGy | 1.0609305758944045 |
| 180 mGy | 0.9886468260044778 |
### Chart: BAK1
| Category | Day 6 |
|---|---|
| 30 mGy | 1.083367030065964 |
| 180 mGy | 1.0568878996359605 |Supplemental Fig S2 | Altered expression of proliferation and apoptosis related genes. Fold changes of gene expression in RNA-seq on day 6 by 30 and 180 mGy. To understand the reduction of EB size (Fig 1B), induced fold changes of four genes under low-dose irradiation in RNA-seq (fpkm) were shown. Marker of proliferation Ki-67 (MKI67), BCL2 associated X, apoptosis regulator (BAX), BCL2 antagonist/killer 1 (BAK1), and Caspase 3 (CASP3) were not changed on day 6 (N=3, Student’s paired t-test).
Disease and function mode of IPA revealed the activation of organismal death (Fig 2B). From these findings, we speculate that there were some other changes, such as protein modifications and following cell cycle arrest or cell death.

## Slide 4
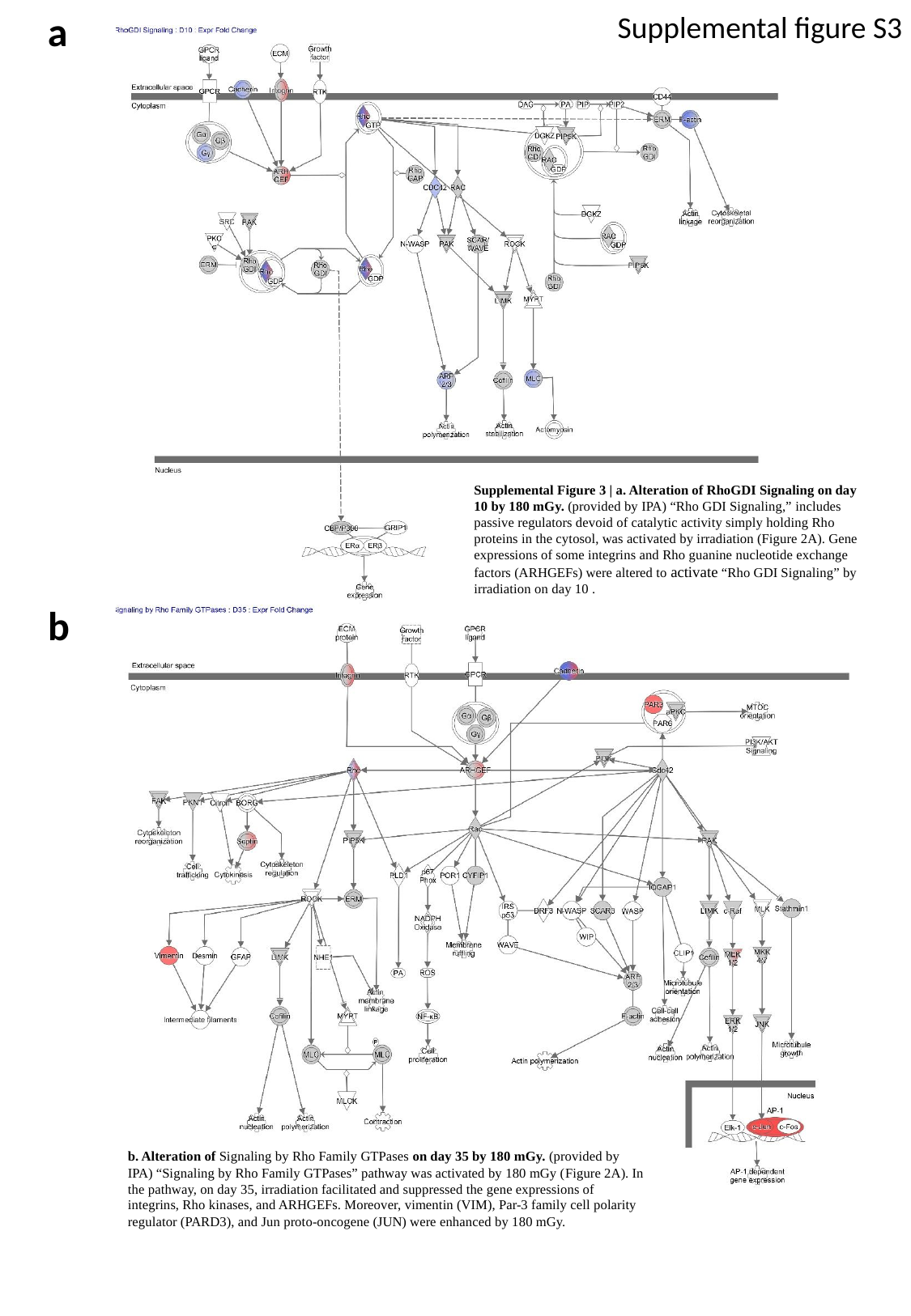

a
Supplemental figure S3
Supplemental Figure 3 | a. Alteration of RhoGDI Signaling on day 10 by 180 mGy. (provided by IPA) “Rho GDI Signaling,” includes passive regulators devoid of catalytic activity simply holding Rho proteins in the cytosol, was activated by irradiation (Figure 2A). Gene expressions of some integrins and Rho guanine nucleotide exchange factors (ARHGEFs) were altered to activate “Rho GDI Signaling” by irradiation on day 10 .
b
b. Alteration of Signaling by Rho Family GTPases on day 35 by 180 mGy. (provided by IPA) “Signaling by Rho Family GTPases” pathway was activated by 180 mGy (Figure 2A). In the pathway, on day 35, irradiation facilitated and suppressed the gene expressions of integrins, Rho kinases, and ARHGEFs. Moreover, vimentin (VIM), Par-3 family cell polarity regulator (PARD3), and Jun proto-oncogene (JUN) were enhanced by 180 mGy.

## Slide 5
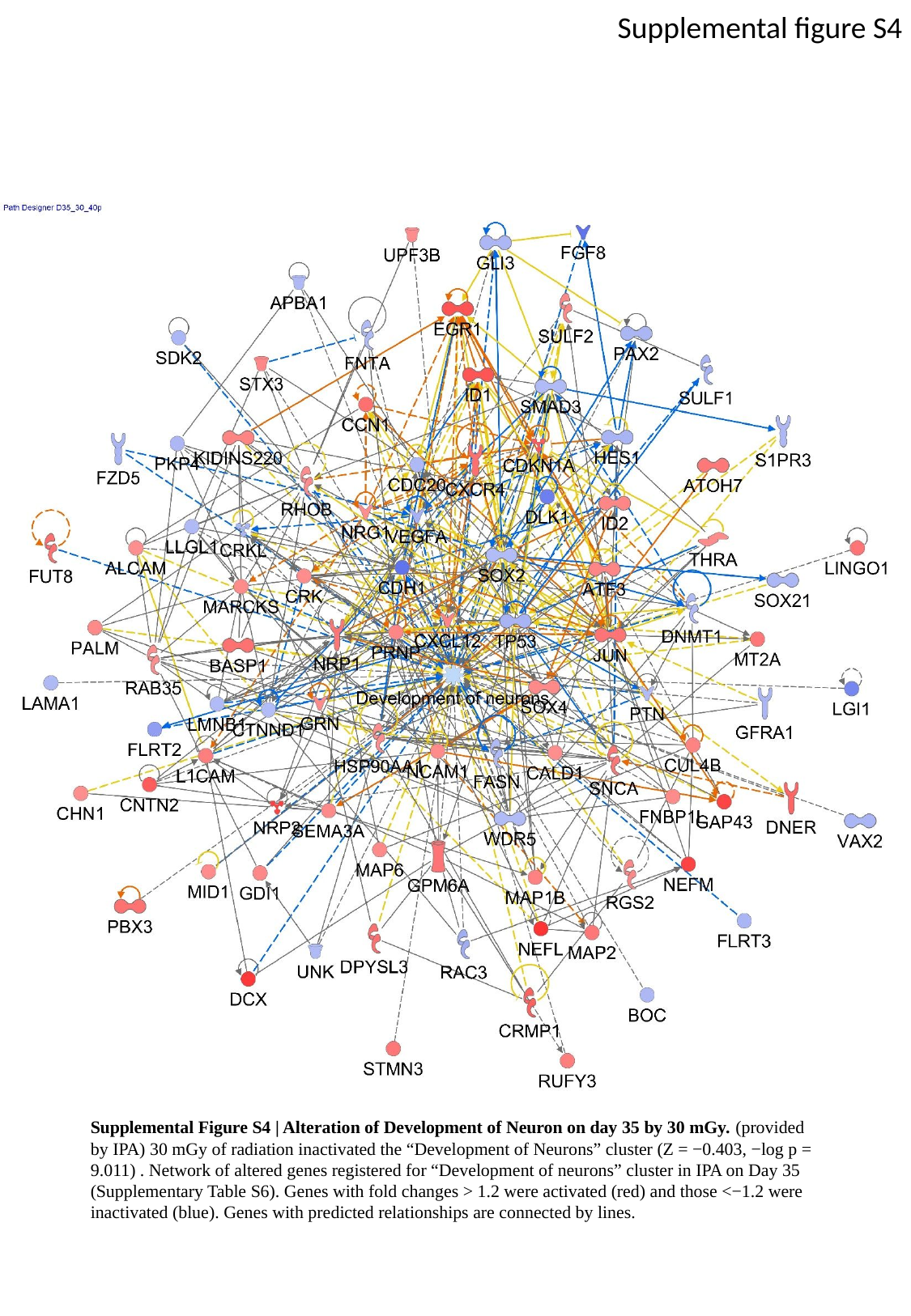

Supplemental figure S4
Supplemental Figure S4 | Alteration of Development of Neuron on day 35 by 30 mGy. (provided by IPA) 30 mGy of radiation inactivated the “Development of Neurons” cluster (Z = −0.403, −log p = 9.011) . Network of altered genes registered for “Development of neurons” cluster in IPA on Day 35 (Supplementary Table S6). Genes with fold changes > 1.2 were activated (red) and those <−1.2 were inactivated (blue). Genes with predicted relationships are connected by lines.

## Slide 6
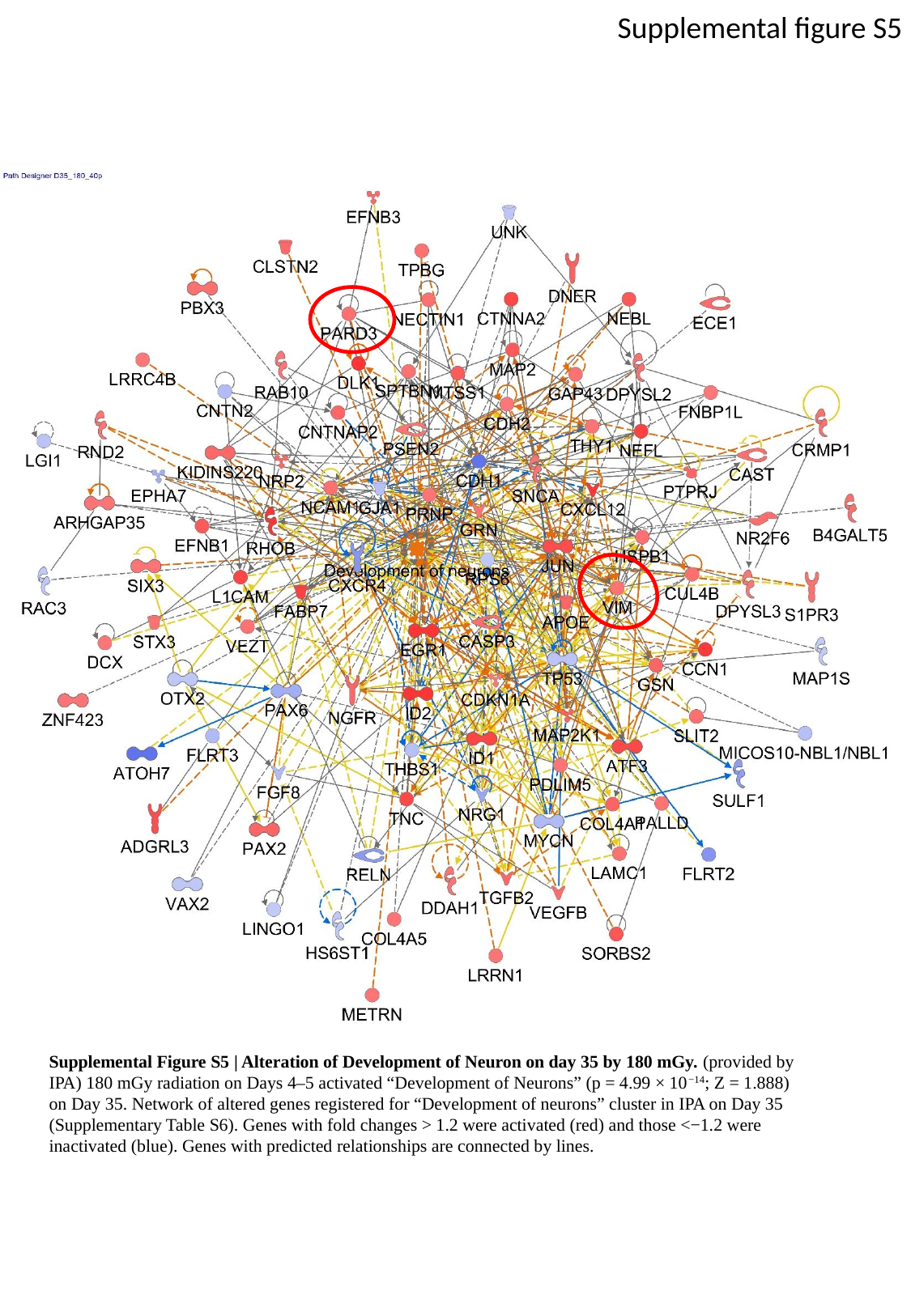

Supplemental figure S5
Supplemental Figure S5 | Alteration of Development of Neuron on day 35 by 180 mGy. (provided by IPA) 180 mGy radiation on Days 4–5 activated “Development of Neurons” (p = 4.99 × 10^−14^; Z = 1.888) on Day 35. Network of altered genes registered for “Development of neurons” cluster in IPA on Day 35 (Supplementary Table S6). Genes with fold changes > 1.2 were activated (red) and those <−1.2 were inactivated (blue). Genes with predicted relationships are connected by lines.

## Slide 7
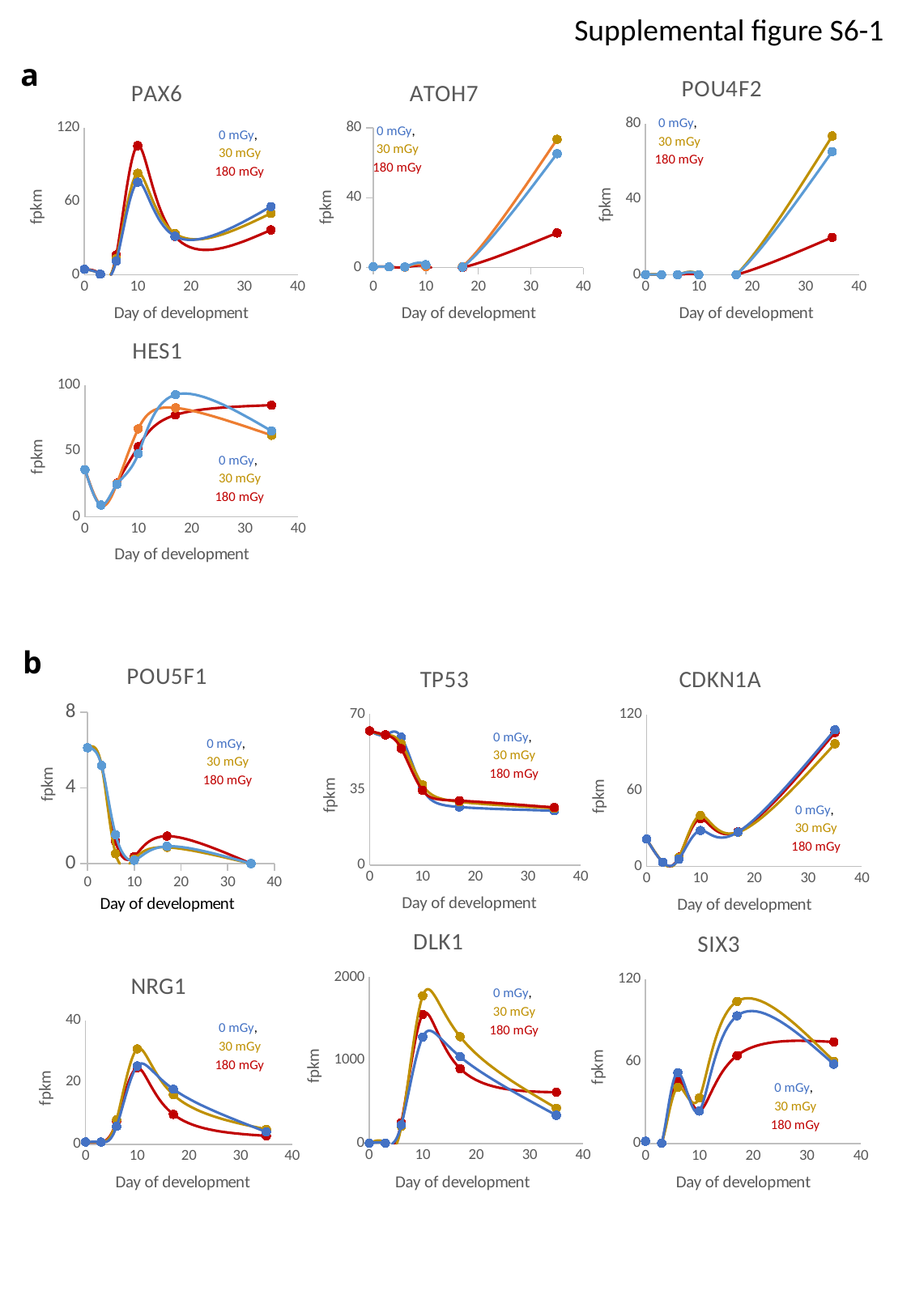

Supplemental figure S6-1
a
### Chart: POU4F2
| Category | | | |
|---|---|---|---|
### Chart: PAX6
| Category | 0mGy | 30mGy | 180mGy |
|---|---|---|---|
### Chart: ATOH7
| Category | | | |
|---|---|---|---|0 mGy,
30 mGy
180 mGy
0 mGy,
30 mGy
180 mGy
0 mGy,
30 mGy
180 mGy
### Chart: HES1
| Category | 0mGy | 30mGy | 180mGy |
|---|---|---|---|0 mGy,
30 mGy
180 mGy
b
### Chart: TP53
| Category | 0mGy | 30mGy | 180mGy |
|---|---|---|---|
### Chart: POU5F1
| Category | 0mGy | 30mGy | 180mGy |
|---|---|---|---|
### Chart: CDKN1A
| Category | 0mGy | 30mGy | 180mGy |
|---|---|---|---|0 mGy,
30 mGy
180 mGy
0 mGy,
30 mGy
180 mGy
0 mGy,
30 mGy
180 mGy
### Chart: DLK1
| Category | 0mGy | 30mGy | 180mGy |
|---|---|---|---|
### Chart: SIX3
| Category | 0mGy | 30mGy | 180mGy |
|---|---|---|---|
### Chart: NRG1
| Category | 0mGy | 30mGy | 180mGy |
|---|---|---|---|0 mGy,
30 mGy
180 mGy
0 mGy,
30 mGy
180 mGy
0 mGy,
30 mGy
180 mGy

## Slide 8
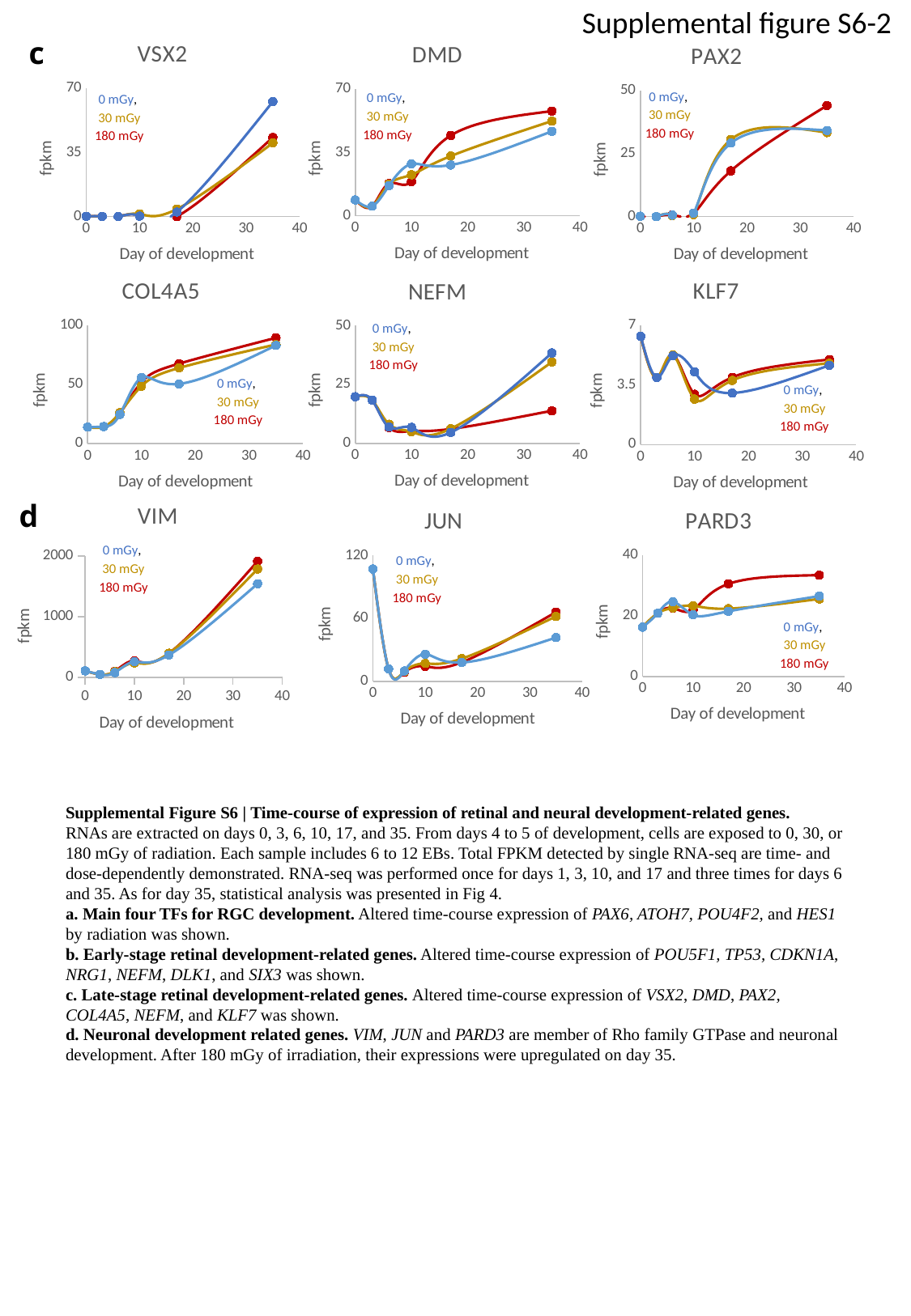

Supplemental figure S6-2
### Chart: VSX2
| Category | 0mGy | 30mGy | 180mGy |
|---|---|---|---|
### Chart: DMD
| Category | 0mGy | 30mGy | 180mGy |
|---|---|---|---|
### Chart: PAX2
| Category | 0mGy | 30mGy | 180mGy |
|---|---|---|---|c
0 mGy,
30 mGy
180 mGy
0 mGy,
30 mGy
180 mGy
0 mGy,
30 mGy
180 mGy
### Chart: COL4A5
| Category | 0mGy | 30mGy | 180mGy |
|---|---|---|---|
### Chart: KLF7
| Category | 0mGy | 30mGy | 180mGy |
|---|---|---|---|
### Chart: NEFM
| Category | 0mGy | 30mGy | 180mGy |
|---|---|---|---|0 mGy,
30 mGy
180 mGy
0 mGy,
30 mGy
180 mGy
0 mGy,
30 mGy
180 mGy
### Chart: VIM
| Category | 0mGy | 30mGy | 180mGy |
|---|---|---|---|d
### Chart: PARD3
| Category | 0mGy | 30mGy | 180mGy |
|---|---|---|---|
### Chart: JUN
| Category | 0mGy | 30mGy | 180mGy |
|---|---|---|---|0 mGy,
30 mGy
180 mGy
0 mGy,
30 mGy
180 mGy
0 mGy,
30 mGy
180 mGy
Supplemental Figure S6 | Time-course of expression of retinal and neural development-related genes.
RNAs are extracted on days 0, 3, 6, 10, 17, and 35. From days 4 to 5 of development, cells are exposed to 0, 30, or 180 mGy of radiation. Each sample includes 6 to 12 EBs. Total FPKM detected by single RNA-seq are time- and dose-dependently demonstrated. RNA-seq was performed once for days 1, 3, 10, and 17 and three times for days 6 and 35. As for day 35, statistical analysis was presented in Fig 4.
a. Main four TFs for RGC development. Altered time-course expression of PAX6, ATOH7, POU4F2, and HES1 by radiation was shown.
b. Early-stage retinal development-related genes. Altered time-course expression of POU5F1, TP53, CDKN1A, NRG1, NEFM, DLK1, and SIX3 was shown.
c. Late-stage retinal development-related genes. Altered time-course expression of VSX2, DMD, PAX2, COL4A5, NEFM, and KLF7 was shown.
d. Neuronal development related genes. VIM, JUN and PARD3 are member of Rho family GTPase and neuronal development. After 180 mGy of irradiation, their expressions were upregulated on day 35.

## Slide 9
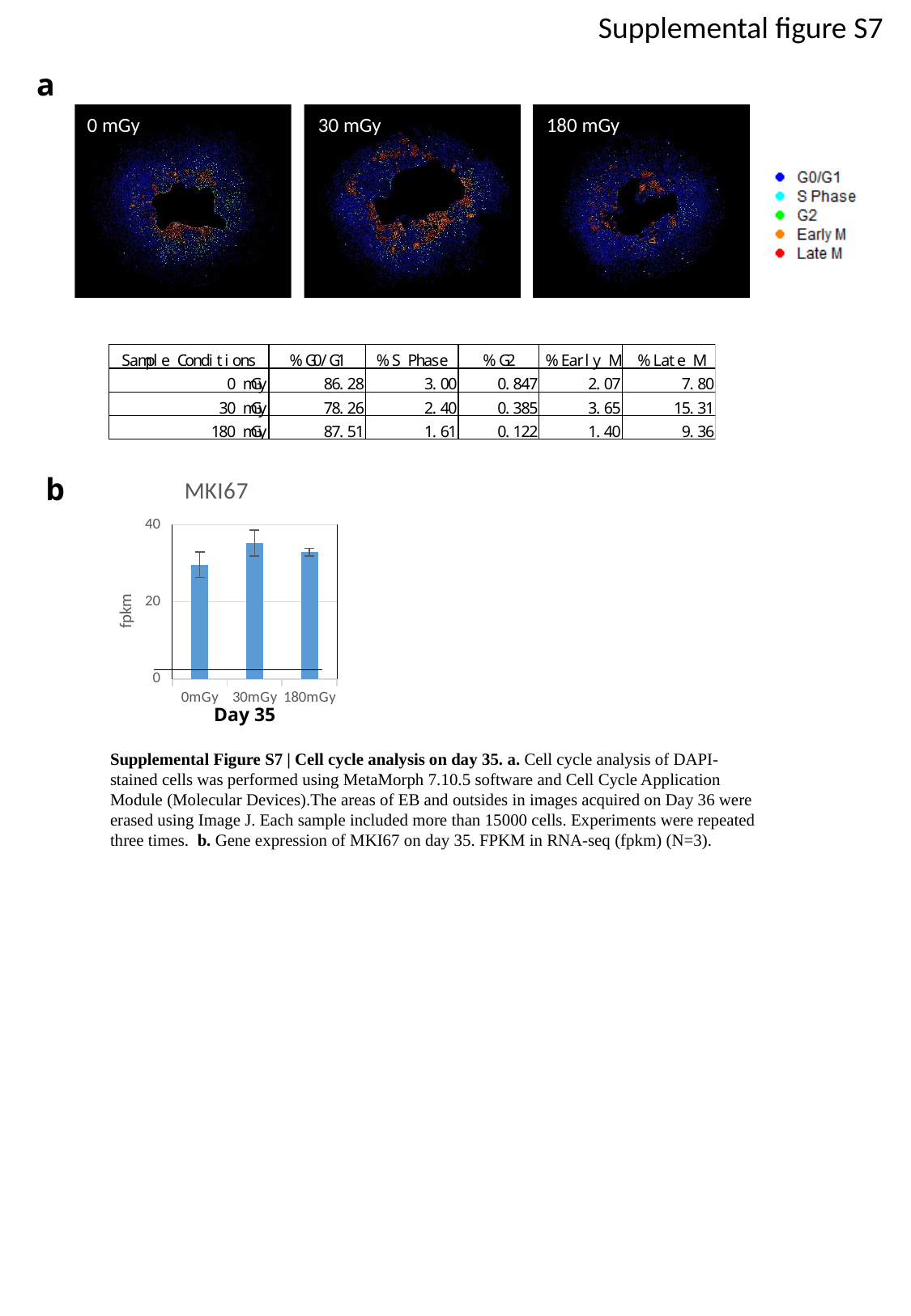

Supplemental figure S7
a
0 mGy
30 mGy
180 mGy
### Chart:
| Category | MKI67 |
|---|---|
| 0mGy | 29.669466666666665 |
| 30mGy | 35.213766666666665 |
| 180mGy | 32.888866666666665 |Day 35
b
Supplemental Figure S7 | Cell cycle analysis on day 35. a. Cell cycle analysis of DAPI-stained cells was performed using MetaMorph 7.10.5 software and Cell Cycle Application Module (Molecular Devices).The areas of EB and outsides in images acquired on Day 36 were erased using Image J. Each sample included more than 15000 cells. Experiments were repeated three times. b. Gene expression of MKI67 on day 35. FPKM in RNA-seq (fpkm) (N=3).
